# Prioritizing transcriptomic and epigenomic experiments using an optimization strategy that leverages imputed data

**DOI:** 10.1093/bioinformatics/btaa830

**Published:** 2020-09-23

**Authors:** Jacob Schreiber, Jeffrey Bilmes, William Stafford Noble

**Affiliations:** Paul G. Allen School of Computer Science and Engineering; Department of Electrical and Computer Engineering; Department of Genome Sciences, University of Washington, Seattle, WA 98195, USA

## Abstract

**Motivation:**

Successful science often involves not only performing experiments well, but also choosing well among many possible experiments. In a hypothesis generation setting, choosing an experiment well means choosing an experiment whose results are interesting or novel. In this work, we formalize this selection procedure in the context of genomics and epigenomics data generation. Specifically, we consider the task faced by a scientific consortium such as the National Institutes of Health ENCODE Consortium, whose goal is to characterize all of the functional elements in the human genome. Given a list of possible cell types or tissue types (‘biosamples’) and a list of possible high-throughput sequencing assays, where at least one experiment has been performed in each biosample and for each assay, we ask ‘Which experiments should ENCODE perform next?’

**Results:**

We demonstrate how to represent this task as a submodular optimization problem, where the goal is to choose a panel of experiments that maximize the *facility location* function. A key aspect of our approach is that we use imputed data, rather than experimental data, to directly answer the posed question. We find that, across several evaluations, our method chooses a panel of experiments that span a diversity of biochemical activity. Finally, we propose two modifications of the facility location function, including a novel submodular–supermodular function, that allow incorporation of domain knowledge or constraints into the optimization procedure.

**Availability and implementation:**

Our method is available as a Python package at https://github.com/jmschrei/kiwano and can be installed using the command pip install kiwano. The source code used here and the similarity matrix can be found at http://doi.org/10.5281/zenodo.3708538.

**Supplementary information:**

Supplementary data are available at *Bioinformatics* online.

## 1 Introduction

Experimental characterization of the genomic and epigenomic landscape of a human cell line or tissue (‘biosample’) is expensive but can potentially yield valuable insights into the molecular basis for development and disease. Fully measuring the epigenome involves, in principle, assaying chromatin accessibility, transcription, dozens of histone modifications and the binding of over a thousand DNA-binding proteins. Even after accounting for the decreasing cost of high-throughput sequencing, such an exhaustive analysis is expensive, and systematically applying such techniques to diverse cell types and cell states is simply infeasible. Essentially, we cannot afford to fill in an experimental data matrix in which rows correspond to types of assays and columns correspond to biosamples.

Several approaches have been proposed to address this challenge. Some scientific consortia, such as GTEx and ENTEX, aim to completely fill in a smaller submatrix of preselected assays and biosamples. In contrast, other consortia, such as the Roadmap Epigenomics Mapping Consortium (Kundaje *et al.*, 2015) and ENCODE (ENCODE Project Consortium, 2012), adopted a roughly ‘L’-shaped strategy, in which consortium members focused on carrying out many assays in a small set of high-priority biosamples, and a smaller set of assays over a much larger set of biosamples. Recently, computational approaches have been proposed that rely on using machine learning models to impute the experiments that have not yet been performed (Durham *et al.*, 2018; Ernst and Kellis, 2015; Schreiber *et al.*, 2020a,b). Although the imputation strategy can relatively easily complete the entire matrix, a drawback is that the imputed data is potentially less trustworthy than actual experimental data.

In this work, we address a variant of the matrix completion problem where the goal is to identify a set of *k* values within the matrix that should be filled in. This problem corresponds to the scenario that we, as a field, find ourselves in currently, where we have performed many assays in many biosamples and are trying to figure out which of the remaining assay/biosample combinations (‘experiments’) we should perform next. However, this problem is difficult because, by definition, we do not know how informative an experiment will be before it is performed. As a result, the choice of which experiments to perform is frequently driven by intuition and guesswork.

A computational approach to this problem is to frame the selection as a submodular optimization problem. In this formulation, a submodular set function *f*(·) quantifies the quality of a panel of assays relative to the full collection of potential experiments, and maximization of this function involves identifying a panel of high quality. Framing the problem as a submodular optimization has several advantages, including principled greedy approaches to optimization, and the ability to seed these greedy algorithms with experiments that have already been performed. However, a challenge facing this optimization is that, generally, *f* relies on calculating similarities between all pairs of input elements, and similarities cannot be calculated for experiments that have not been performed.

Wei *et al.* (2016) overcome this challenge by restricting panel selection to one axis of the experimental matrix at a time. Although this restriction does not allow similarities to be calculated when assays are missing for the given biosample (or biosamples for a given assay), these similarities can be inferred by calculating the average similarity for each pair of assays across all other biosamples where both assays have been performed. After calculating this assay × assay similarity matrix, they choose a panel of assays by using standard methods to maximize a ‘facility location’ set function. An important consequence of calculating similarities in this manner is that their method is ‘biosample-agnostic,’ in the sense that it yields a single set of suggested assays, irrespective of biosample. Wei *et al.* explicitly consider the scenario in which a specified set of assays has already been performed in a given biosample, and the task is to select the next *k* assays to perform. However, even in this setting, the proposed approach is cell-type agnostic: the method yields the same answer for any biosample in which the specified set of assays has been performed.

In this work, we overcome the challenge of calculating similarities for experiments that have not yet been performed by making use of imputed data. Because imputed data exists for every experiment, there is no need to aggregate similarities across biosamples, as Wei *et al.* do. Rather, a panel of experiments can be chosen directly by maximizing the facility location function using the experiment × experiment similarity matrix. This approach has two advantages over the work of Wei *et al.* First, rather than restricting our selection to a single row or column of the data matrix, using imputed data allows us to address the global question, ‘Among all possible experiments within the experimental matrix, which one should I do next?’ Second, even in the case where we want to choose a panel of assays within a single given biosample, our imputation-based approach selects a set that is tailored to this particular biosample.

We use ENCODE data to validate our approach in several ways. First, we illustrate via visualization that the imputation-based similarity matrix encodes meaningful biological relationships among assay types and biosamples that are matched by the real data. We then apply the optimization procedure to this similarity matrix and show that the resulting subset of experiments is representative of the full set, both qualitatively and through simulation experiments. In these simulation experiments, we find that our approach outperforms the approach of Wei *et al.* in a setting similar to the one that they consider. Next, although many of our experiments involve optimizing a plain facility location function, we show that one can include weights and a novel supermodular regularization term to incorporate domain knowledge that is important for choosing a practical set of experiments. Finally, we illustrate how to apply the objective function used in our optimization to ascertain which biosamples are currently undercharacterized and which assays are underutilized. We have made a tool available at https://www.github.com/jmschrei/kiwano/ that can order experiments based on the pre-calculated similarity matrix we use here.

## 2 Materials and methods

### 2.1 Submodular optimization and facility location

Submodular optimization is the discrete analog of convex optimization and operates on submodular set functions. A function is submodular if and only if it has the property of diminishing returns; i.e. the incremental gain in function value associated with adding an element s to a set A becomes smaller as the size of the set A becomes larger. More formally, given a finite set $S=\{s_{1},s_{2},\ldots ,s_{n}\}$, a discrete set function $f:2^{S}\to \mathbb{R}$ is submodular if and only if

f(A∪s)−f(A)≥f(B∪s)−f(B),∀A⊆B⊂S,s∉B.

In this work, we use a submodular function whose value is inversely related to the redundancy within a given set. Thus, optimizing such a function, subject to a cardinality constraint, involves identifying the subset whose elements are minimally redundant with each other. For further reading on submodular optimization, we suggest Fujishige (2005), Krause and Golovin (2014) and Lovász (1983).

Our method relies on optimizing a particular submodular function called facility location. Facility location takes the form

(1) $$f(X)=\sum_{y\in Y}\underset{x\in X}{\max}\phi (x,y)$$

such that *Y* is the full set of experiments, *X* is the selected subset of experiments such that X⊆Y, *x* and *y* are individual experiments in *X* and *Y* respectively, and ϕ(x,y) is the squared correlation between *x* and *y*. The facility location function is optimized using the accelerated greedy algorithm (Minoux, 1978), which iteratively selects the experiment that increases the gain by the largest amount. We use apricot v0.3.0 to perform this selection (Schreiber *et al.*, 2019).

### 2.2 Model training

We evaluated our selection process by training multi-task linear regression models using selected tracks as input and the full set of tracks as the output. These models were implemented using keras (v2.2.4) (Chollet *et al.*, 2015) with a Theano (v1.0.4) (Theano Development Team, 2016) backend. The weight matrix that transformed inputs to outputs was optimized using the Adam optimizer (Kingma and Ba, 2015) with a mean-squared-error loss. All hyperparameters and the weight initializations are set to the keras defaults with no explicit regularization.

These models were trained and evaluated using different partitions of the ENCODE Pilot Regions. First, when the experiments to use were selected using submodular optimization, they were selected using the similarity matrix based on the first 600 000 25 bp bins used throughout this work. Second, the models were trained using the next 500 000 25 bp bins. Finally, the models were evaluated on the remaining 99 362 25 bp bins. There is no overlap between these three partitions.

### 2.3 Datasets

We generated our imputations using an Avocado model that had previously been trained on the ENCODE2018-Core dataset (Schreiber *et al.*, 2020a). The model is available at https://noble.gs.washington.edu/ jmschr/mango/models/. This model was trained on 3814 experiments across 400 biosamples and 84 assays where the signal was $-{\text{log}}_{10}$  *P*-values that had subsequently been arcsin*h* transformed to reduce the effect of outliers. The resulting imputations are in the same space. Due to the large size of the genome, we only imputed the ENCODE Pilot Regions, comprising ∼1% of the genome (ENCODE Project Consortium, 2007), for each of the 33 600 potential experiments. This 1% is comprised of a handful of manually selected regions that were deemed of particular biological interest, combined with a randomly selected set of 30 1-Mb regions that systematically vary in terms of gene density level of non-exonic conservation.

An important detail is that, at the time of accession, experiments measuring transcription had been divided into plus-strand signal and minus-strand signal on the ENCODE portal. Consequently, each strand was counted as a separate assay when training the Avocado model. While the strand that transcription occurs on is important for an imputation approach to capture, this distinction is not helpful for prioritizing experiments because one generally cannot perform an experiment measuring transcription on only one of the strands. Thus, we combine the plus- and minus-strand experiments for both the imputed and the primary epigenomic data by simply adding the tracks together. This process reduced the total number of assays from 84 to 77, the total number of performed experiments from 3814 to 3510, and the total number of potential experiments from 33 600 to 30 800.

## 3 Results

### 3.1 Imputations cluster according to known biological patterns

Our approach for prioritizing experimental characterization relies on a similarity matrix that is calculated on imputed experiments. To produce this matrix, we first generated imputations of epigenomic and transcriptomic experiments using a recently developed imputation approach based on deep tensor factorization, named Avocado. These imputations span 400 human biosamples and 77 assays of biological activity for a total of 30 800 imputed tracks. After acquiring these imputations, we calculated the squared Pearson correlation between all pairs of imputed experiments for use as a similarity measure, resulting in a 30 800 by 30 800 matrix. For efficiency, these correlations were computed with respect to the ENCODE Pilot Regions (ENCODE Project Consortium, 2007), comprising 1% of the genome.

**Fig. 1.**
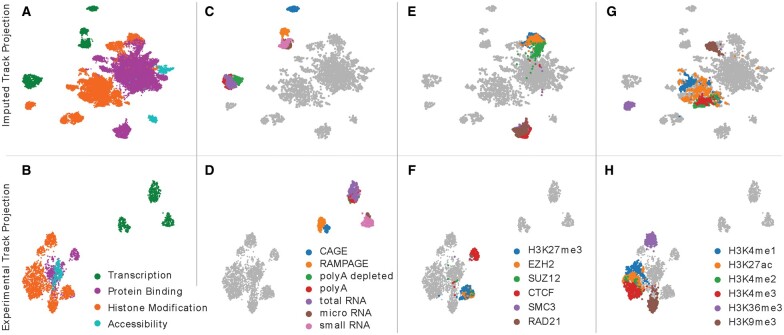
A projection of imputed and experimental epigenomic tracks. Each panel shows a UMAP projection of 30 800 imputed experiments (top row) or of 3150 tracks of primary data (bottom row). In each column, a different set of experiments is highlighted based on their biological activity. (**A**/**B**) Experiments are highlighted based on broad categorization of the assayed activity. (**C**/**D**) Transcription-measuring experiments are colored according to different types of assays. (**E**/**F**) Experiments are highlighted that measure H3K27me3 and two polycomb subunits, as well as CTCF and two cohesin subunits. (G/H) Experiments are highlighted showing several histone modifications that are enhancer-associated, such as H3K4me1 (blue) and H3K27ac (orange), promoter-associated such as H3K4me2 (green) and H3K4me3 (red), transcription-associated such as H3K36me3 (purple) or broadly repressive such as H3K9me3 (brown)

After calculating the similarity matrix, we investigated whether the similarity matrix was able to capture high level biological trends that would be crucial for prioritization. We began by visually inspecting a two-dimensional UMAP projection (McInnes and Healy, 2018) of the similarity matrix down to two dimensions (Fig. 1A). The clearest trend in this projection is a separation of experiments based on a broad categorization of the type of activity measured by the assay. We observed that one cluster contained mostly protein binding experiments, one contained mostly histone modification experiments, and several neighboring clusters were composed exclusively of transcription-measuring experiments. Initially, one might expect that experiments in the same biosample where the assays measure the same underlying phenomena might cluster together. However, we observed that in some cases a pair of experiments may exhibit low correlation when the shape of their signals along the genome differ, even when the assays used in the experiments both measure the same underlying biological activity. For example, the histone modification H3K36me3 is known to be associated with transcription but generally forms broad peaks across the entire gene body, whereas assays such as CAGE or RAMPAGE form punctate peaks.

To confirm that the separation according to assay categorization was not an artifact of the imputation process, we used the same process to calculate a similarity matrix and subsequent UMAP projection for the 3150 tracks of the experimental (or ‘primary’) data (Fig. 1B). The major trends present in the projection of imputed data are consistent with those in the primary data. In particular, transcription experiments form distinct clusters, protein binding experiments are mostly distinct from histone modification ones, and chromatin accessibility experiments localize closer to protein binding experiments than to histone modification experiments. Note that although the figure may appear to show that accessibility experiments overlap with protein binding experiments, a closer examination reveals that the protein binding experiments mostly surround the accessibility experiments.

Next, we more closely examined four sets of assays that, *a priori*, we expected to show distinctive patterns. The first set of experiments was those that measured transcription. When we highlighted experiments by assay type, we observed CAGE and RAMPAGE experiments forming distinct cluters, micro- and small-RNA-seq experiments forming a third cluster, and polyA-, polyA-depleted- and total-RNA-seq experiments forming a fourth (Fig. 1C and D). The second and third sets of experiments involved triplets of assays whose activity are usually associated, specifically, with CTCF and the cohesin subunits, SMC3 and RAD21, as well as H3K27me3 and two polycomb subunits, EZH2 and SUZ12 (Fig. 1E and F). In both cases we observe distinct clusters of experiments, which is particularly interesting for H3K27me3 and the polycomb subunits because one assay measures a histone modification and the other two measure protein binding. The fourth set of experiments focused on six well-studied histone modifications (Fig. 1G and H). The clustering of these six marks coincides with the genomic element in which they are typically enriched. In particular, experiments measuring H3K36me3 and H3K9me3 form their own clusters, with the two assays respectively measuring activity enriched in gene bodies and constitutive heterochromatin. Further, the primary cluster of histone modification experiments exhibited a separation between the promoter-associated marks, H3K4me2 and H3K4me3, and the enhancer-associated marks, H3K4me1 and H3K27ac. We observed similar patterns across both the imputed and primary data for each of these four sets of assays. Taken together, these observations suggest that a similarity matrix derived from imputed experiments is successfully capturing important aspects of real biological activity.

### 3.2 Submodular selection of imputations flexibly prioritizes assays across cellular contexts

Having shown that the similarity matrix captures several high-level trends in the data, we turn to the task of experimental prioritization. Our strategy for prioritizing experiments relies on submodular selection, which is a technique for reducing a set of elements to a minimally redundant subset through the optimization of a submodular function that captures the quality, or ‘representativeness,’ of a given subset relative to the full set (see Section 2 for details). Submodular selection has been used previously to select genomics assays (Wei *et al.*, 2016), to select representative sets of protein sequences (Libbrecht *et al.*, 2018) and to choose genomic loci for characterization by CRISPR-based screens (Gasperini *et al.*, 2019). Specifically, we optimize a ‘facility location’ objective function, which operates on pairwise similarities between elements and so is well suited to leverage our similarity matrix (see Section 2). A critical property of submodular functions is that greedy optimization will yield a subset whose objective value is within $1-e^{-1}$ of the optimal subset, and that this is the best approximation one can make unless P=NP (Nemhauser *et al.*, 1978). This greedy optimization algorithm iteratively selects the single element whose inclusion in the representative set leads to the largest gain in the objective function. Thus, when applied to our similarity matrix, the submodular selection procedure will yield an ordering over all experiments that attempts to minimize redundancy among those experiments that are selected early in the process.

**Fig. 2.**
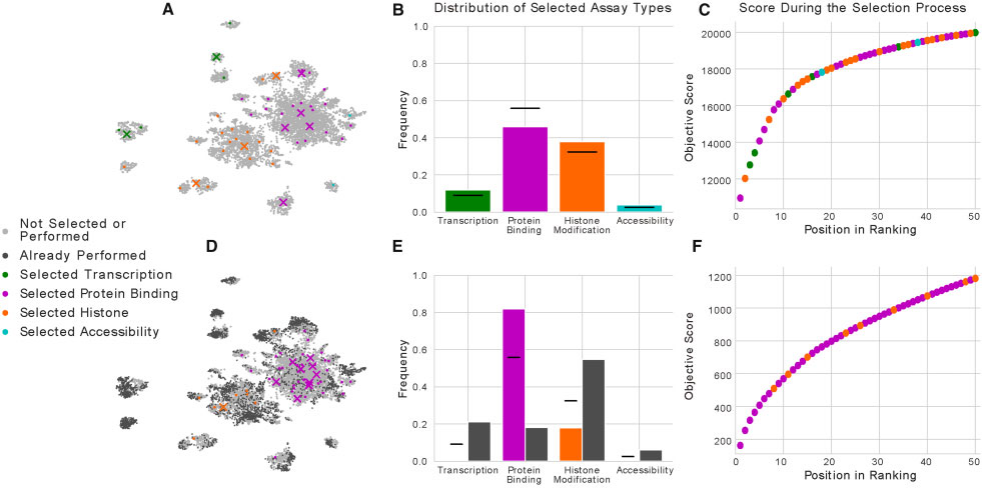
A selection of experiments before and after accounting for those that have already been performed. (**A**) The same projection of imputed experiments as shown in Figure 1A, where the first 50 experiments selected using our method are colored by the type of activity that they measure. The first 10 experiments selected are marked using an X, and the remaining 40 are marked with a dot. (**B**) A bar chart showing the frequency that experiments of each type of activity are selected in the first 50 experiments. (**C**) The facility location objective score as the first 50 experiments are selected, with each point colored by the type of activity measured by that experiment. (**D**) The same as (A), but with the selection procedure initialized with the experiments that have already been performed, and with those experiments displayed in dark gray. (**E**) The same as (B), but with dark gray bars showing the frequency of experiments of each type that have already been performed. (**F**) The same as (C), but with the selection procedure initialized with the experiments that have already been performed

To demonstrate that submodular selection results in a representative subset of assays, we applied it to our calculated similarity matrix. Visually, we observe that the first 50 selected experiments appear to cover the space well and include selections from many of the small clusters of experiments (Fig. 2A, Supplementary Table S1). When we count the number of assays selected for each type of biological activity, we find that protein binding assays are the most commonly selected with 23 experiments, followed by histone modification assays with 19 experiments, transcription assays with 6 experiments, and, finally, accessibility assays with 2 experiments (Fig. 2B). However, when we compare the number of selected experiments of each type to the number that one would expect by randomly selecting with replacement, we observe that protein binding experiments are underrepresented, whereas histone modification experiments are overrepresented. We note that the first 10 experiments are at the centers of large clusters of experiments and that the subsequent 40 experiments are selected from smaller clusters. This finding corresponds with the gain in the facility location objective score from each successive experiment significantly diminishing by the tenth experiment (Fig. 2C).

We then evaluated the stability of our procedure by applying it to randomly selected sets of genomic loci drawn from the entire genome, rather than a portion of the ENCODE Pilot Regions. As a practical matter, we could not select these positions from the imputations themselves because it would involve storing >30k genome-wide tracks in memory. Furthermore, because each track took roughly half a gigabyte to store in memory, we were restricted to using only a subset of 800 of the 3150 experimental tracks. When we applied our procedure to these 800 tracks to choose 50 experiments, we found that a similar number of experiments were selected across 100 runs for each type of biological activity (Supplementary Fig. 1A). Likewise, we found a similar number of experiments selected per assay (Supplementary Fig. 1B). We also noted that these numbers were similar to the number of experiments chosen when using the ENCODE Pilot Regions. Together, these results indicate that the selection is stable to small perturbations in the correlation matrix, and that the ENCODE Pilot Regions are a reasonable approximation of a random sample of the entire genome.

Next, we consider whether the selection process is influenced by the number of available experiments for each biosample or assay. Potentially, biosamples and assays with plentiful available data will have more information-rich imputations that are consequently prioritized highly by our method, whereas biosamples and assays with little available data will have poor quality imputations that appear similar to each other and are largely ignored by the method. However, we did not find any evidence that this was the case. When we used our approach to choose a panel of 500 experiments, we observed little correlation between the number of available experiments and the number of chosen experiments for each biosample and assay (Supplementary Fig. 2). Further, we found that only 5.6% (28) of the 500 chosen experiments corresponds to experiments that had actually been performed and were used to train the underlying imputation model, indicating that our method does not simply select experiments from the training set of the underlying imputation model.

A weakness in simply applying submodular selection to the full set of imputed experiments is that the procedure does not account for the thousands of epigenomic and transcriptomic experiments that have already been performed. Fortunately, there are two ways that one can account for these experiments. The first is to remove those experiments that have already been performed from the similarity matrix and perform selection on the remaining experiments. While this approach is simple, it does not account for the content of the experiments that have already been performed. For example, if transcription has already been measured in hundreds of biosamples, then it may be beneficial to focus experimental efforts on characterizing other types of biological activity. A second approach takes advantage of the fact that the selection process is greedy by initializing the set of selected experiments with those that have already been performed. This ensures that the selected experiments cover types of activity that are not already well characterized.

Accordingly, we proceeded with the second approach. We initialized a facility location function with the 3150 experiments that had already been performed and ranked the remaining 27 650 experiments. We observed that the selected experiments lie primarily in areas of the UMAP projection that do not already have many experiments performed (Fig. 2D, Supplementary Table S2). When we counted the number of selected experiments of each type, we found that the number of protein binding experiments increased from 23, when not accounting for the experiments that had already been performed, to 41, when accounting for them (Fig. 2E). Correspondingly, the number of histone modification experiments decreased from 19 to 9. This change in coverage is likely because 1726 experiments measuring histone modification have already been performed, whereas only 571 experiments measuring protein binding have been performed. Further, none of the first 50 selected experiments measure transcription or accessibility, likely because those forms of activity are already much better measured than protein binding. In this setting, the gain in the facility location objective function of each successive experiment is much lower, due in large part to the experiments that have already been performed (Fig. 2F).

### 3.3 Selection on imputed experiments identify diversity in primary data

Our next step was to evaluate the quality of the selected experiments in a quantitative way. Following Wei *et al.*, we reasoned that the signal contained in a representative subset of experiments would be well suited for reconstructing the signal in all experiments. We formulated the problem of quantitatively measuring how representative a subset is as a multi-task regression problem, with the input features being the signal from the selected subset of experiments and the outputs being the signal from the full set of experiments (see Section 2). Importantly, to ensure that this validation measured how representative a subset is of the primary data, despite subset selection having been performed on the imputations, we used the primary data as both the input and target for this task.

We selected a subset of experiments in three ways. The first was through the submodular selection procedure described in Section 3.2, applied to the 3150 imputed experiments for which primary data had already been collected. The second was by applying the submodular selection procedure to the 3150 tracks of primary data themselves. Naturally, selecting subsets based on the primary data cannot be extended to experiments that have not yet been performed, and so the purpose of evaluating models trained using this subset is to measure the effect that the imputation process itself has on selecting a representative subset of experiments. The third was selecting subsets of the 3150 performed experiments at random. This random process was repeated 20 times to obtain a distribution of scores.

**Fig. 3.**
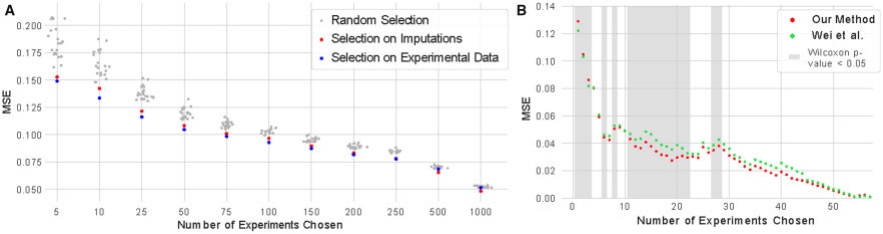
Imputation performance using different panels of assays. (**A**) The performance of regression models (in terms of mean-squared-error, MSE) as a function of the number of experiments chosen as the input. These panels range in size from 5 assays to 1000 assays, and are selected either randomly (gray), through a facility location function applied to imputed experiments (red), or through a facility location function applied to primary data (blue). (B) The average performance of regression models across all biosamples as a function of the number of experiments chosen as the input for that biosample. The panels are chosen either using our approach or the method of Wei et al. Gray shading indicates differences that are significant at Wilcoxon *P*-value ≤0.05

We observed that the subsets of experiments selected using submodular selection consistently outperform those selected at random (Fig. 3A). Each comparison is statistically significant at a *P*-value threshold of 0.01 according to a one sample *t*-test. Further, for smaller subsets, applying submodular selection to the imputed tracks performs nearly as well as the panels selected on the primary data itself, showing that the distortion introduced by the imputation process is small. Interestingly, when the subsets become much larger, those selected using imputed tracks appear to outperform those selected using the primary data. This trend may arise because imputed tracks can serve as denoised versions of the primary data (Ernst and Kellis, 2015). At the beginning of the selection process, this denoising is not necessary to select experiments that are very different from each other. However, once many experiments have been selected, the denoised experiments may be better at identifying real differences between experiments. We observed similar trends when the selection step was performed on a different set of experiments from those used to evaluate the regression model (Supplementary Fig. ??A), and when an entire form of biochemical activity, in this case protein binding assays, was held out from the selection step and then used to evaluate the regression model (Supplementary Fig. ??B).

Next, we compared our approach to the approach proposed by Wei *et al.* in their restricted setting of choosing a subset of assays within a particular biosample. Specifically, for each biosample, we derive similarity matrices from the imputed experiments for that biosample (our approach) or the average similarity over experiments from every other biosample (their approach). It is worth mentioning that, even though this evaluation setting is similar to the one considered by Wei *et al.*, it is not identical: our approach is still restricted to the set of biosamples where imputations are available. In a similar manner to the previous evaluations in this section, we then evaluate how well regression models can use varying sized subsets of assays from a particular biosample to predict the full set of assays within the biosample. The errors are then averaged across biosamples for each panel size.

In this setting, our method outperformed the method of Wei *et al.* for most subset sizes, and frequently by a large margin (Fig. 3B). Although our method results in errors up to 5.6% higher for subsets of sizes one through three, it achieves reductions in MSE of up to 27.4% for larger panel sizes. Further, our approach achieves a lower error with a Wilcoxon *P*-value ≤0.05 for 16 panel sizes (Wei *et al.* outperforms our method with a Wilcoxon *P*-value ≤0.05 for panel sizes between one and three). We noted that most panel sizes do not show a statistically significant difference between the two methods, mostly because there are few biosamples where a large number of assays have been performed. These results indicate that the use of imputed data leads to better panels of experiments than averaging similarities across other biosamples.

### 3.4 Incorporating domain information by extending the objective function

At this point, we have demonstrated that our procedure can choose a panel of experiments that exhibit diverse functional activity. However, because the procedure considers only the calculated similarity matrix when making choices, the resulting panel of experiments may be suboptimal in practice. One reason for this is that some experiments can be easily integrated with other sources of information, enhancing their overall usefulness. For example, chromatin accessibility measurements from DNase-seq or ATAC-seq can be paired with genomic sequence to identify protein binding motifs or other regulatory sequences. Another reason that the panel may be suboptimal is that the chosen experiments are likely to be *scattered* across a large number of biosamples or assays. Although this property is desirable in theory, preparing many biosamples or acquiring the materials to perform many different types of assays can be difficult or expensive in practice.

The facility location objective function can be modified to incorporate a weight for each experiment that encourages or discourages the selection of those experiments. These weights provide a straightforward way to incorporate estimates of the relative utility of each experiment, when such is known in advance. The weighted facility location objective function is

(2) $$f(X)=\sum_{y\in Y}\underset{x\in X}{\max}w(x)\phi (x,y),$$

where *w* is a function that returns a non-negative weight of experiment *x*. Because this weight does not change during the optimization process, this weighted objective remains submodular and can be optimized in the same manner as the original objective.

**Fig. 4.**
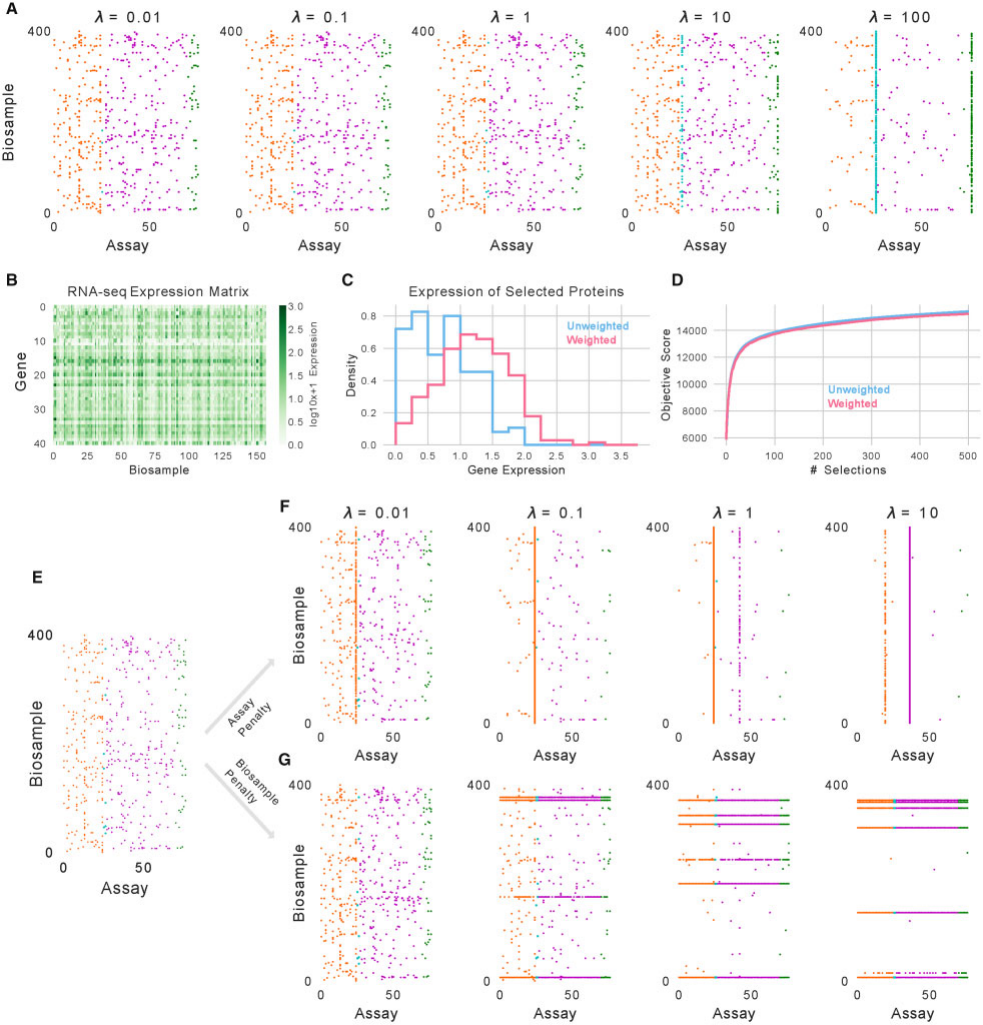
Controlling selection with an extended objective function. (**A**) The 500 experiments selected as the weights for DNase and total RNA-seq experiments changes. (**B**) Log-transformed gene expression values for the proteins whose binding is assayed in ENCODE2018-Core across 157 biosamples. (**C**) The expression values for selected protein binding experiments in the original setting (blue) and when weighting experiments by the log-transformed expression values (pink). (**D**) The score from the facility location objective function without weighting from the experiment subsets when weighting or not weighting the objective. (**E**) A selection of 500 experiments performed normally. (**F**) A selection of 500 experiments when mixing the submodular function with a supermodular assay penalty of varying weight. (**G**) A selection of 500 experiments when mixing the submodular function with a supermodular biosample penalty of varying weight

Using this objective, we explored the effect that the weights had on the chosen experiments. We began by varying the weights for all DNase-seq experiments from 0.01 to 100, setting the weights for all total RNA-seq experiments to half that of the DNase-seq weight, and keeping the weights of all other experiments at 1. We then selected 500 experiments in the same manner as our initial selection experiments for each of five weights for DNase-seq experiments (Fig. 4A). As expected, a small weight of 0.01 resulted in a panel that did not contain DNase-seq or total RNA-seq experiments at all, whereas a weight of 100 resulted in a panel that predominately consisted of these experiments, with 222 DNase-seq experiments and 137 total RNA-seq experiments. These results demonstrate not only that the weights influence the selection process but that the relative weights are important, with roughly half as many total RNA-seq experiments selected as DNase-seq experiments.

We then considered a more sophisticated use of the weights by setting them using biological measurements. A potential issue with our selection of protein binding experiments is that not all proteins are expressed in every biosample (Fig. 4B). A protein that is not expressed cannot bind to the genome, and the corresponding ChIP-seq experiment measuring binding of that protein, although yielding a flat signal that is likely very dissimilar to other protein binding experiments, would be uninformative. We can penalize the selection of such unwarranted experiments by weighting protein binding experiments using gene expression values from RNA-seq assays. Specifically, we set the weight of each protein binding assay to $\;{\text{log}}_{10}(\mathrm{TPM}+1)$ where *TPM* is the transcripts-per-million value from a total RNA-seq experiment for the relevant gene and biosample. This weighting scheme downweights protein binding experiments when the protein is not expressed in that biosample and upweight those experiments where the protein is expressed highly. Because some biosamples did not have a total RNA-seq experiment performed in them, we excluded from this analysis all protein binding experiments from biosamples without a total RNA-seq experiment performed in it.

We find that weighting experiments in this manner results in choosing fewer unwarranted experiments. Overall, this procedure raised the median TPM of the genes encoding the assayed proteins from 4.1 to 16.2 and reduced the number of chosen experiments with a corresponding TPM of <0.5 from 22 to 4 (Fig. 4C). Interestingly, we did not find that using a weighted objective to choose a panel of experiments resulted in experiments with a significantly lower score according to the original objective function: the panel chosen from the weighted function achieved 98.8% of the score of the panel chosen using the original function (Fig. 4D).

Next, we considered a further modification to the objective function that reduces the scatter of chosen experiments across the experimental matrix. This modification involves adding to the facility location function a pair of regularization terms that count the number of experiments in each biosample or assay. More formally, we define functions *a* and *b* that take in a set of experiments and return a vector of the count of experiments that involve each assay or biosample, respectively. We incorporate these terms into a new objective function

(3) $$f(X)=\sum_{y\in Y}\underset{x\in X}{\max}w(x)\phi (x,y)+\lambda_{a}||a(Y)|{|}_{2}^{2}+\lambda_{b}||b(Y)|{|}_{2}^{2},$$

where *λ_a_* is a weight that encourages experiments to span fewer assays and *λ_b_* is a weight that encourages experiments to span fewer biosamples. This function is not submodular because $\lambda_{a}||a(Y)|{|}_{2}^{2}$ and $\lambda_{b}||b(Y)|{|}_{2}^{2}$ grow during the selection process and, thus, violate the diminishing returns property. Although the greedy algorithm does not have the same guarantees when applied submodular–supermodular mixtures as it does on purely submodular functions, it has been shown to perform well empirically (Bai and Bilmes, 2018).

In a similar set of experiments as when we weighted DNase-seq and total RNA-seq experiments, we next inspected the selections made as we varied *λ_a_* and *λ_b_*. As expected, we see that as we increase the regularization strength the selected experiments span fewer assays and biosamples, respectively (Fig. 4E–G). Interestingly, we observed that as the regularization strength increased from $\lambda_{a}=1$ to $\lambda_{a}=10$ the most chosen assay switched from measuring the histone modification H4K91ac to the binding of ETS1. This finding suggests that utility of a particular assay is dependent on the number of other experiments that can be performed to supplement it.

### 3.5 Calculating the coverage of each biosample and assay

Thus far, we have focused our efforts on prioritizing individual experiments but have provided little guidance for how to prioritize entire biosamples or assays. We next considered a scenario where an investigator is looking to either assay undercharacterized biosamples or to run underperformed assays, but is unsure which biosamples or assays to focus on. A simple approach would be to count the number of experiments that each biosample or assay is involved in and choose the ones with the fewest experiments. However, this approach does not account for the content of the performed experiments, which can be extremely similar in some cases. For example, in the ENCODE data several biosamples have been assayed extensively for transcription but not assayed at all for histone modifications or protein binding.

A final component of our methodology is the ability to quantify the extent to which each biosample has been characterized and each assay has been performed using the facility location objective function. Because the objective function takes in a set of experiments and returns a score corresponding to the diversity of the set, this function can be used to assess the diversity obtained by an existing set of experiments, corresponding to a single biosample or a single type of assay. In our setting, where similarity is measured via squared correlation, this score ranges from zero up to the total number of experiments that have been performed. Thus, for each biosample, the maximum value is 77 due to the 77 assays in the dataset, and for each assay, the maximum value is 400 due to the 400 biosamples in the dataset.

We applied this approach to score each of the biosamples and assays in the ENCODE2018-Core dataset. Not surprisingly, we find that the three ENCODE Tier 1 cell lines—H1-hESC, K562 and GM12878—are the three best scoring biosamples, with scores of 71.2, 70.2 and 68.6, respectively. These biosamples are followed by several ENCODE Tier 2 cell lines, such as HepG2, IMR90 and HeLa-S3. We found a rank correlation of 0.82 between the number of assays performed in a biosample and the objective score, confirming that while in general there is increase in coverage as more assays are performed, the composition of those assays is also captured by the objective function. Next, we scored the assays and found that the highest scoring ones were H3K4me3, H3K36me3 and CTCF, whereas the lowest scoring assays are H2BK15ac and FOXK2. We found a weaker, but still very significant, rank correlation of 0.66 between the number of biosamples that an assay was performed in and the objective score.

**Fig. 5.**
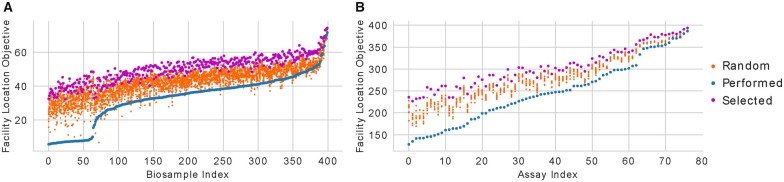
Scoring biosamples and assays according to their captured diversity. (**A**) The facility location objective score for each biosample when applied to the set of experiments that investigators have performed in that biosample (blue), the set of experiments identified by optimizing the objective function (magenta), and the sets of randomly selected experiments (orange), ordered by the score of the performed experiments. (**B**) The same as (A), but for each assay instead of each biosample

We next sought to contextualize the scores we obtained for each biosample and assay by comparing them to scores obtained if one had used alternate methods to select experiments. For each element, i.e. a particular assay or biosample, we scored 10 randomly selected panels of the same size as the number of experiments involving that element. In addition, we score the panel of experiments that would have been selected using submodular selection. We observe a striking result, which is that the set of experiments that were actually performed not only underperforms the set selected through submodular selection, but also generally underperform random selection (Fig. 5). This trend is consistent across both biosamples and assays. We note that the 64 biosamples with the worst scores were assayed almost exclusively for transcription, supporting the notion that biosamples with more assays performed in them are not always better characterized.

## 4 Discussion

In this work, we describe an approach for the prioritization of epigenomic and transcriptomic experiments that has the potential to increase the rate of scientific discovery by focusing characterization efforts on those experiments that are expected to yield the least redundant information. To our knowledge, this is the first approach that enables the global prioritization of experiments across both biosamples and assays. We anticipate that, due to the time it takes to perform experiments and the simplicity of our method, investigators may use our prioritization methods even when they plan eventually to perform all potential experiments to begin analyses sooner.

An important consideration is that, due to the reliance on imputed experiments, our method cannot be applied directly to a biosample or assay type when no experiments have yet been performed. In particular, in the setting where a researcher aims to characterize a distinct system for which essentially nothing is known about the different biosamples and assays, then it would be necessary to first perform a subset of experiments that include all assays and biosamples for use in training an imputation model, and then use the resulting imputations to prioritize the remaining experiments. Potentially, for such a system, it may be possible to identify closely related experiments for which imputations have already been generated. Although these imputations may not capture activity specific to an experiment, it is likely that the resulting similarity matrix would provide a reasonable approximation. However, in the setting where one truly knows nothing about a biosample or assay, the method proposed by Wei *et al.* provides a principled approach for choosing an initial panel of assays to comprehensively perform in each biosample in the system before imputing the remaining missing experiments.

Although the primary question we address is how to prioritize experiments across both biosamples and assays, we recognize that this approach may not always result in a practical set of experiments to perform. In practice, it is generally more difficult to culture and maintain a variety of biosamples than it is to maintain a large quantity of a single biosample, making sets of experiments that span several biosamples harder to perform than those in the same biosample. This difficulty may cause investigators to prefer performing batches of experiments within a biosample. Accordingly, we have proposed weighting experiments to incorporate domain knowledge and a novel submodular–supermodular function that encourages the chosen experiments to span a compact set of biosamples and assays. However, there are likely more aspects of performing experiments, e.g. the predicted cost of each experiment and the anticipated difficulty, that could also be directly included in the optimization procedure.

When we scored the biosamples in the ENCODE2018-Core dataset using the facility location objective function, we noted that the actual set of assays performed in many biosamples performed worse than randomly selecting an equally sized panel of assays. However, this trend is not entirely surprising. The experiments that are included in our dataset were intentionally devised to investigate specific research questions, and generally these questions do not aim to broadly characterize the human epigenome. Thus, these results serve primarily to demonstrate that the current strategy for selecting experiments is not well suited for the goal of characterizing the overall diversity of activity in the human epigenome.

A potential weakness in our method is that mistakes in the imputation process may be propagated to the selection process. These mistakes can be simple errors in predicting certain peaks or can involve more systematic trends. For example, REST is a transcription factor that is involved in suppressing neuronal genes in non-neuronal tissues. However, the ENCODE2018-Core dataset does not have examples of REST in neuronal tissue, and so an imputation model trained on this dataset would likely be unaware of this property of REST. Consequently, the prioritization process is unlikely to capture that REST binding in neuronal tissues is significantly different than in non-neuronal tissues. Another consideration is that our method may be unlikely to prioritize experiments in biosamples with poor quality imputations because those imputations are usually similar to the average signal, i.e. appear to be unsurprising. In general, unexpected patterns in data that has not yet been collected will be difficult for any prioritization method to account for.

The flexibility of our method allows for several extensions that we did not consider here, but may nonetheless prove valuable to those prioritizing experiments. The first is that, in the setting where one is prioritizing experiments within a particular biosample, one could measure the gain that each successive experiment adds to the objective function to determine when to stop performing experiments. This would serve as a data-driven indicator of when further experimental efforts are mostly redundant. A second extension is that one could calculate the similarity matrix using only a specific genomic locus or set of loci of interest. For example, if an investigator was aiming to experimentally quantify the activity surrounding an important gene across many biosamples, one could restrict the similarity calculation to a window surrounding that gene. Overall, our approach is a simple yet powerful way to prioritize experiments in a wide variety of contexts.

## Supplementary Material

btaa830_Supplementary_Data
