# Supplementary material for: Prioritizing transcriptomic and epigenomic experiments using an optimization strategy that leverages imputed data
Source: Bioinformatics. 2020 Sep 23;37(4):439–47. doi: 10.1093/bioinformatics/btaa830 (PMC8088321; doi:10.1093/bioinformatics/btaa830)
Supplement: btaa830_Supplementary_Data [file btaa830_supplementary_data.zip › supplement.pdf]

Supplement

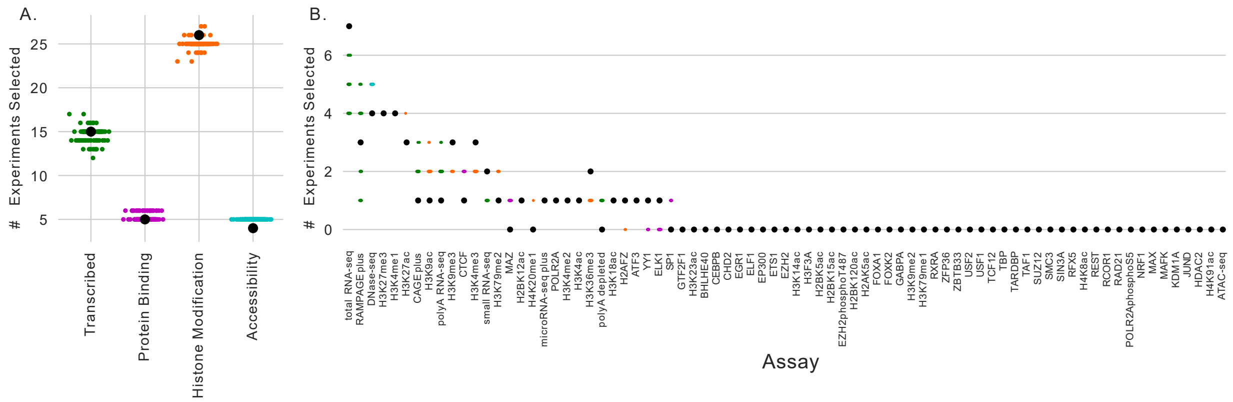

Figure S1: **Selection stability over 100 random subsets of positions.** The number of experiments selected across 100 random subsets of positions and using the ENCODE Pilot Regions (in black) (A) for each type of biochemical activity and (B) for each individual assay.

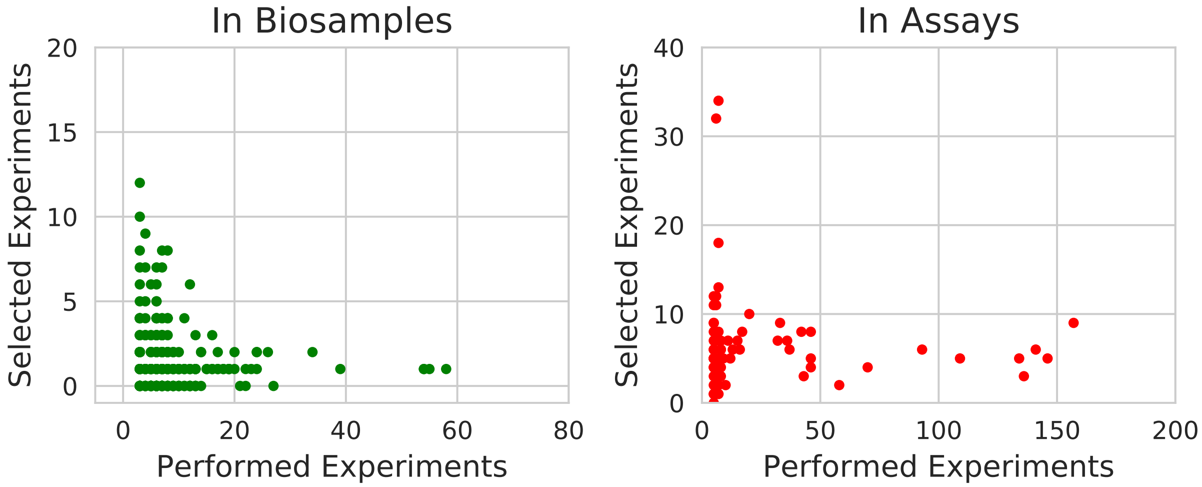

Figure S2: **Counts of available and selected experiments.** The number of available and selected experiments when choosing a panel of 500 experiments partitioned by biosample (left) and assay (right).

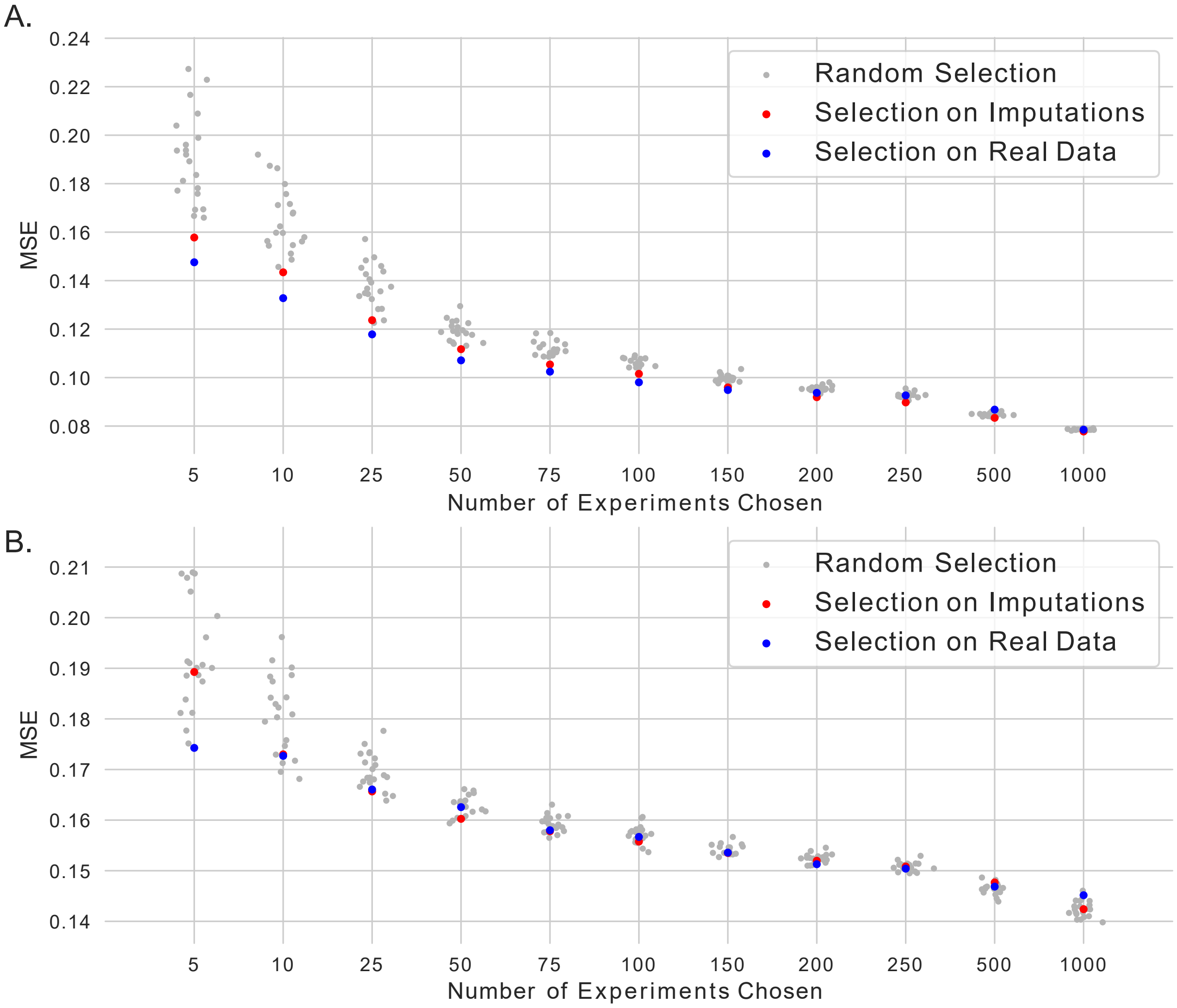

Figure S3: **Predictive performance of models trained using selected experiments.** (A) Performance of machine learning models trained using varying numbers of experiments as input that are selected from one half of all experiments and evaluated on the other half of all experiments. (B) Similar to A except the models are evaluated solely on predicting protein binding experiments and the input experiments are selected from all other experiments.
